# Application of machine learning to explore the genomic prediction accuracy of fall dormancy in autotetraploid alfalfa

**DOI:** 10.1093/hr/uhac225

**Published:** 2022-10-07

**Authors:** Fan Zhang, Junmei Kang, Ruicai Long, Mingna Li, Yan Sun, Fei He, Xueqian Jiang, Changfu Yang, Xijiang Yang, Jie Kong, Yiwen Wang, Zhen Wang, Zhiwu Zhang, Qingchuan Yang

**Affiliations:** Institute of Animal Science, Chinese Academy of Agricultural Sciences, Beijing, China, 100193; Department of Crop and Soil Sciences, Washington State University, Pullman, WA, USA, 99163; Institute of Animal Science, Chinese Academy of Agricultural Sciences, Beijing, China, 100193; Institute of Animal Science, Chinese Academy of Agricultural Sciences, Beijing, China, 100193; Institute of Animal Science, Chinese Academy of Agricultural Sciences, Beijing, China, 100193; Department of Turf Science and Engineering, College of Grassland Science and Technology, China Agricultural University, Beijing, China, 100193; Institute of Animal Science, Chinese Academy of Agricultural Sciences, Beijing, China, 100193; Institute of Animal Science, Chinese Academy of Agricultural Sciences, Beijing, China, 100193; Institute of Animal Science, Chinese Academy of Agricultural Sciences, Beijing, China, 100193; Institute of Animal Science, Chinese Academy of Agricultural Sciences, Beijing, China, 100193; Institute of Animal Science, Chinese Academy of Agricultural Sciences, Beijing, China, 100193; Melbourne Integrative Genomics, School of Mathematics and Statistics, University of Melbourne, Melbourne, Australia, 3052; Institute of Animal Science, Chinese Academy of Agricultural Sciences, Beijing, China, 100193; Department of Crop and Soil Sciences, Washington State University, Pullman, WA, USA, 99163; Institute of Animal Science, Chinese Academy of Agricultural Sciences, Beijing, China, 100193

## Abstract

Fall dormancy (FD) is an essential trait to overcome winter damage and for alfalfa (*Medicago sativa*) cultivar selection. The plant regrowth height after autumn clipping is an indirect way to evaluate FD. Transcriptomics, proteomics, and quantitative trait locus mapping have revealed crucial genes correlated with FD; however, these genes cannot predict alfalfa FD very well. Here, we conducted genomic prediction of FD using whole-genome SNP markers based on machine learning-related methods, including support vector machine (SVM) regression, and regularization-related methods, such as Lasso and ridge regression. The results showed that using SVM regression with linear kernel and the top 3000 genome-wide association study (GWAS)-associated markers achieved the highest prediction accuracy for FD of 64.1%. For plant regrowth height, the prediction accuracy was 59.0% using the 3000 GWAS-associated markers and the SVM linear model. This was better than the results using whole-genome markers (25.0%). Therefore, the method we explored for alfalfa FD prediction outperformed the other models, such as Lasso and ElasticNet. The study suggests the feasibility of using machine learning to predict FD with GWAS-associated markers, and the GWAS-associated markers combined with machine learning would benefit FD-related traits as well. Application of the methodology may provide potential targets for FD selection, which would accelerate genetic research and molecular breeding of alfalfa with optimized FD.

## Introduction

Fall dormancy (FD) refers to the adaptive growth characteristic of alfalfa in autumn and can be evaluated by plant regrowth height at 25–30 days after final cutting [1]. A taller alfalfa plant indicates a lower degree of dormancy. The FD level of alfalfa varieties can be classified from 1 to 11, where 1 represents the most dormant and 11 the least dormant [1]. The main inducers of dormancy are falling temperature and a shortening photoperiod. Dormancy can improve plant survival in winter and increase freezing tolerance. FD cultivars usually have better winter survival and hardiness than non-dormant cultivars [2–5]. FD level is one of the criteria for alfalfa cultivar selection in specific regions. There is a trade-off between FD and yield. High FD increases overwintering ability but decreases the potential yield [5, 6]. Studying the genetic architecture of FD could be used to improve those high-yield alfalfa cultivars with weak cold tolerance [7, 8].

Multi-omics methods, including genomics, transcriptomics, and proteomics, are efficient in revealing the genetic mechanism of FD. With quantitative trait locus (QTL) mapping, some crucial FD-related QTLs have been found using different FD-level alfalfa germplasms in several studies. For instance, Li *et al*. have identified 71 QTLs related to FD traits, and 15 QTLs were detected in more than one environment [7]. Another study identified 45 significantly associated QTLs for FD [9]. A gene expression-related experiment showed that a cold acclimation-responsive gene, *RootCAR1*, was positively associated with alfalfa winter survival [10]. Using transcriptomics technology, FD-related genes have been found. For example, 44 genes were identified by comparing non-FD with FD cultivars’ leaves. These genes include *IAA-amino acid hydrolase ILR1-like 1*, *abscisic acid receptor PYL8*, and *monogalactosyldiacylglycerol synthase-3* [11]. Eight significantly differentially expressed transcriptional factors related to C-repeat binding factors (CBF) and ABA-responsive elements (ABRE) were identified [12]. FD-related proteins were analyzed using proteomics and metabolomics. A total of 90 proteins were found to be different between FD and non-FD alfalfa. One of these is the thiazole biosynthetic enzyme (*MsThi*), which is essential for alfalfa growth [13]. Raffinose family oligosaccharide (RFO) was found to be involved in short photoperiod-induced freezing tolerance in dormant alfalfa cultivars [14].

Genome-wide association studies (GWAS) help precisely locate the single-nucleotide polymorphisms (SNPs) associated with the corresponding phenotypes using historical recombination information. With the development of sequencing and genetic analysis methods of GWAS, in several alfalfa populations a set of candidate SNP markers were identified to be responsible for critical quantitative traits [15–17]. Based on 198 accessions, a set of GWAS results have been reported, including 19 SNPs associated with drought resistance [18], 36 SNPs related to salt tolerance [17], 42 associated with plant growth and forage production [19], and 131 markers associated with 26 forage quality traits [20]. Using 336 genotypes, GWAS was conducted for several key alfalfa traits, including fiber-related traits and digestibility [16], crude protein and mineral concentrations [21], and nine biomass-related traits [22]. Several SNPs associated with forage quality were identified using half-sib progeny developed from three cultivars [15]. Furthermore, several genes were found to be responsible for some crucial traits of alfalfa with the GWAS method. For example, several stress-responsive genes associated with yield under water deficit were identified, including *leucine-rich repeat receptor-like kinase*, *B3 DNA-binding domain protein*, *translation initiation factor IF2*, and *phospholipase-like protein* [19]. Two markers linked to *NIR-NBS-LRR* genes were found to be significantly associated with *Verticillium* wilt resistance [23]. A cell wall biosynthesis gene associated with several cell wall-related traits that affect alfalfa nutritional value has been identified [24]. *MsACR11* affects plant height, which significantly increases the size of transgenic arabidopsis [22].

Genomic prediction (GP, or genomic selection) is an efficient way to use whole-genome markers to predict crop trait performance without prior knowledge about trait-associated genes [25]. Some progress has been made in alfalfa breeding-related GP. For example, The prediction accuracy of yield was 0.32–0.35 in two genetically contrasting populations using genotyping-by-sequencing (GBS) markers [26]. The predicted accuracy of yield can be achieved as high as 0.4 using cross-population validation [27]. With the forage quality-related traits, the predicted accuracy is ~0.3–0.4 [15]. The model-building step using the training population is a crucial step for GP. Prediction models, such as Lasso, ridge regression, and machine learning methods, can be used for GP. Machine learning methods have been used recently to predict crop traits [28]. In alfalfa yield, machine learning models can use weather, historical yield, and sown date to make predictions. Published results have shown that the *k*-nearest neighbor and random forest methods performed well [29].

GWAS and GP have been performed in alfalfa and both have achieved promising results, but few studies have combined GWAS and GP to predict traits. In addition, sequencing technology and prediction methods restricted GP accuracy in previous studies. In this study, we evaluated FD (defined as the plant regrowth height at 30 days after autumn clipping) using GWAS and GP jointly with resequencing data and FD of 220 accessions [1]. The objectives of this experiment were to: (i) compare the predicted accuracy of different GP methods; (ii) calculate FD predicted accuracy using the hybrid model of GWAS and GP; and (iii) extend the GP model for new trait prediction.

## Results

### Phenotypic variance and single-nucleotide polymorphism filter

The variance of the phenotype was analyzed to evaluate the genetic difference in FD for different individuals of an accession. The effects of accession, year, individual, and accession year interaction were estimated (Supplementary Data Table S2). FD was recorded for two years: 2018 and 2019. The FD correlation coefficient between individual mean and the mean of all FD values was 0.69 (Supplementary Data Fig. S2). The variance of FD in 220 accessions was analyzed using ANOVA. Significant differences in accession, year, and accession–year interaction were observed at the probability level of *P* < .001. The variation among individuals was not significant (*P* = .12), indicating that the difference in FD between individuals is not big, and the mean value can be used to represent the FD of an accession (Supplementary Data Table S2). The broad-sense heritability (*h*^2^) was 80.6% for FD.

A total of 32.2 million SNPs were detected using the BWA-SAMtools-VarScan pipeline. After filtering with a missing value >10%, minimum mean read depth >20, and minor allele frequency >0.05, 875 023 SNPs passed the threshold and were confirmed for GWAS analysis. The individual heterozygosity of the 220 accessions ranged from 0.06 to 0.18. The mean heterozygosity was 0.11 (Supplementary Data Fig. S3). To remove the effect of the linked marker within the linkage disequilibrium interval, the linked SNP marker was filtered by a sliding window using PLINK with parameter —indep-pairwise 100 50 0.2. Finally, 431 299 markers passed the threshold and were used for GP analysis. Missing genotypes were imputed with Beagle software [30].

### Prediction of fall dormancy with cross-validation

**Figure 1:**
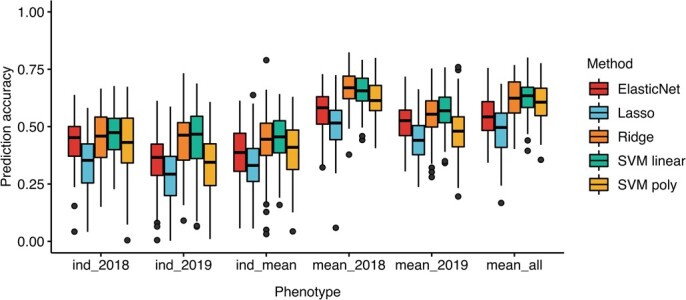
Prediction accuracy across six kinds of FD phenotype using different genomic prediction methods.

Six kinds of the phenotype were used for model evaluation. Three sequencing individual-related phenotypes, i.e. ind_2018, ind_2019 and ind_mean, represented the phenotype of the corresponding sequenced plant in 2018, 2019, and mean values of 2018 and 2019, respectively. The other three kinds of phenotype, i.e. mean_2018, mean_2019 and mean_all, represented the mean FD of 15 individuals in 2018, 2019, and the mean value of all FD data within one accession, respectively. Five prediction methods [ElasticNet, Lasso, ridge regression, support vector machine (SVM) linear, and SVM poly] were compared for prediction accuracy and bias. The average prediction accuracy using the mean of all phenotypes was 62.8% and 61.8% for SVM linear and ridge regression, respectively, indicating that the average accuracy of SVM linear and ridge regression was better than that of the other three models. The mean GP accuracy was higher for SVM linear than ridge regression. The SVM linear method was used for the subsequent analyses. The prediction accuracy of the Lasso method was worse than that of the others. ElasticNet and SVM poly had a medium prediction accuracy. The average prediction accuracy of Lasso, ElasticNet, and SVM poly was 47.7%, 54.2% and 60.7%, respectively. The prediction variance among 100 repeats in six kinds of phenotype is shown in Fig. 1.

**Figure 2:**
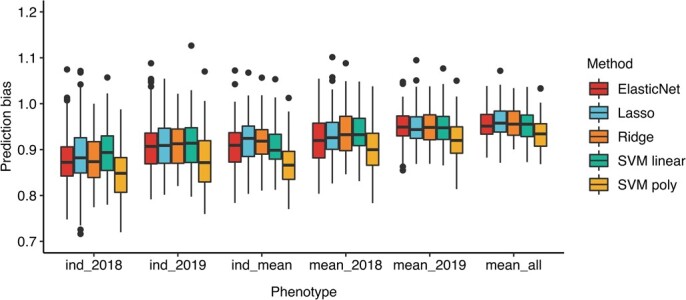
Prediction bias across six kinds FD phenotype using different genomic prediction methods.

Prediction bias was used to estimate inflated or deflated predictions. The results showed that all five methods had deflated predictions (Fig. 2). The average prediction accuracy of ElasticNet, Lasso, ridge regression, SVM linear and SVM poly was 0.95, 0.96, 0.96, 0.95, and 0.93, respectively. The similar prediction bias of these methods (except for SVM poly) suggested that FD traits may be more suitable to fit training models using a linear model than a non-linear model.

### Genome-wide association study for fall dormancy

To identify SNP markers associated with FD and compare the effect of different phenotypes, genotypic and phenotypic data were analyzed using the Bayesian-information and Linkage-disequilibrium Iteratively Nested Keyway (BLINK) method in the BLINK C-version software. Two kinds of phenotype, i.e. mean of all (mean_all) and individual mean (ind_mean), were used to conduct GWAS analysis.

**Figure 3:**
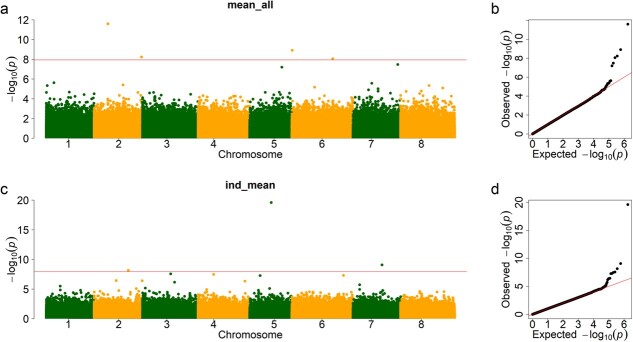
Manhattan and Q–Q plot of FD. The GWAS was performed by BLINK C version software, with a significant *P*-value threshold set at *P* = .01/875023 = 1.14 × 10^–8^ (red line). We identified four significantly associated SNPs of mean of all FD, shown in the Manhattan plot (**a**). Q–Q plots are displayed in the right panel (**b**). **c**, **d** Three associated SNPs of individual mean FD.

The quantile–quantile (Q–Q) plot results of marker–trait associations for FD are illustrated using observed versus expected *P*-values in Fig. 3. Any deviation from the expected red line implies SNP association with FD. In the present study, a total of four SNPs passed the 1% threshold after Bonferroni correction (*P* < 1.14 × 10^–8^) and were associated with FD of mean of all (mean_all) phenotype and three SNPs associated with FD of individual mean (ind_mean) phenotype (Fig. 3). Among them, two markers, chr2__23380918 and chr2__80663885, were located on chromosome 2, and the remaining two markers (chr6__740339 and chr6__69737395) were located on chromosome 6. The three markers associated with FD of individual mean were located on chromosomes 2 (chr2__58174960), 5 (chr5__36702273), and 7 (chr7__49431088) (Supplementary Data Table S3). To get unbiased results for phenotype-associated SNPs, the GWAS-associated SNP was checked using information for separate years. Four associated markers were identified (Supplementary Data Table S3). They may reflect different genetic information on FD.

### Using genome-wide association study-associated markers for fall dormancy prediction

**Figure 4:**
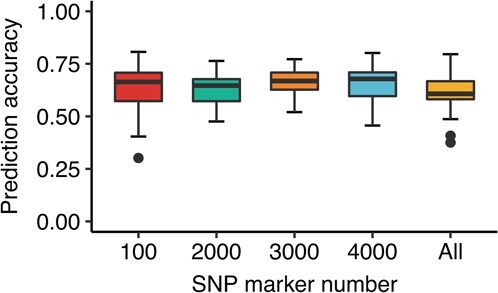
Box plot of mean of all FD prediction accuracies among different numbers of individual mean FD GWAS-associated markers.

To check the individual selection effect for mean phenotype change, the GWAS-associated markers of individual mean FD was used to predict the mean of all phenotypes. Different numbers of associated markers (top 100–10 000) were used to make predictions. The total markers (875 023) were used to check the mean prediction accuracy (Supplementary Data Fig. S4). The results showed that 3000 associated markers had the highest mean GP of 64.1%. When all markers were used to make predictions, the mean GP was 59.6%. The mean GP was 61.1%, 61.9% and 63.0% for 100, 2000 and 4000 associated markers, respectively (Fig. 4). The variance of GP among 100 repeats was the lowest for 3000 associated markers.

### Using fall dormancy-associated markers for fall growth prediction

**Figure 5:**
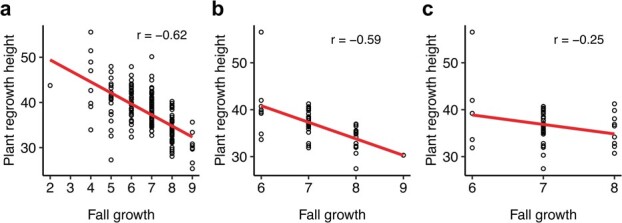
Correlation between fall growth and plant regrowth height for different kinds of fall growth phenotype. Every circular dot represents one accession. The regression line is shown as a red line, and Pearson’s correlation coefficient is shown at the upper right of each panel. **a** Actual fall growth phenotype. **b** Predicted fall growth phenotype using 3000 FD-associated markers. **c** Predicted fall growth phenotype using all 875 023 markers.

GWAS-associated SNP markers were further used to predict a new trait (fall growth). Both kinds of GWAS results (individual mean and mean of all) were used to select prediction markers. The top 1500 markers for individual mean and mean of all GWAS-associated SNPs were combined to get 3000 GP markers. There were 17 repeat markers in both kinds of phenotype. Finally, 2983 markers were selected to make the prediction. Fall growth phenotype downloaded from the GRIN-Global database was used to generate the training phenotype (176 samples), and the remaining 44 samples without fall growth phenotype were predicted using 2983 GWAS markers. Mean plant regrowth height phenotype collected in 2018 and 2019 was used to check prediction accuracy. Negative correlation was found between actual fall growth phenotype and plant regrowth height. Pearson’s correlation coefficient was −0.62 (prediction accuracy 62.0%). The prediction accuracy was 59.0% using the predicted fall growth phenotype. However, the prediction accuracy was 25.0% using all markers (Fig. 5).

## Discussion

### The feasibility of using machine learning methods to predict fall dormancy in alfalfa

Machine learning methods have been widely used in crop phenotypic prediction [28]. One promising direction is using machine learning methods to explore big data from omics technologies. It has been used in polyploid plants, such as strawberry and blueberry [31]. One study applied machine learning methods to predict alfalfa yield. They compared the correlation-based method, ReliefF method, and wrapper method. The results showed that the correlation-based method is better than others. With feature selection, the *k*-nearest neighbor and random forest methods could predict alfalfa yield [29]. In this study, we used five methods to predict FD. Our results showed that ridge regression and SVM linear methods achieved a similar prediction accuracy for FD in different kinds of phenotypes. The prediction bias was close for these two methods. This means both can be used for the prediction of FD in alfalfa. The prediction accuracy of Lasso was the worst, while the accuracy of ElasticNet was between Lasso and ridge regression, indicating feature selection could decrease the prediction accuracy. There should be many minor effects of QTLs to control FD. SVM regression with linear kernel (SVM linear) had higher prediction accuracy than poly kernel (SVM poly), suggesting the linear model has better prediction accuracy than a non-linear model for FD. Other machine learning linear methods, such as random forest (RF) and reproducing kernels Hilbert spaces regression (RKHS), may achieve similar prediction accuracy [28], but the genetic architecture of a particular trait has its own specificity. If one model corresponds to the genetic architecture of a trait, this model will have high prediction accuracy [32]. Taking the results together, for alfalfa FD prediction the SVM linear machine learning method performs exceedingly well.

### The efficiency of genomic prediction using genome-wide associationy study-associated markers

GWAS is a valuable method to identify trait-associated markers in alfalfa genetic-related studies [23, 33]. A total of seven SNPs were associated with FD based on the 1% Bonferroni test (Fig. 4). The broad-sense *h*^2^ estimated was 80.6%, suggesting there are other markers related to FD. We tried the GWAS-associated markers for the prediction of FD. To remove the influence of GWAS-related phenotype in the cross-validation step, the GWAS-associated markers of individual mean FD were used to predict the mean of all phenotypes (from individual FD predicted accession mean FD). The marker number ranged from 100 to 10 000. The results showed that 3000 markers had the highest predicted accuracy, 64.1%, which is 4.5% higher than that of using all markers. Hence, using GWAS-associated markers increased prediction accuracy. The mean accuracy was 64.1% using 3000 markers, meaning that Pearson’s correlation coefficient between the predicted mean of all phenotypes and the actual mean of all phenotypes was 0.64. The actual correlation coefficient between individual mean and mean of all was 0.69 (Supplementary Data Fig. S2). Hence, GWAS-associated markers achieved prediction accuracy similar to that of phenotypic selection.

Our cross-validation prediction accuracy (62.8%) was higher than in most previous studies of alfalfa. For example, the yield and forage quality predicted accuracies were around 30%–40% [15, 26, 27]. There are two possible explanations for the phenomenon. One is heritability. For yield-related traits, the heritability is generally around 50% [34], which restricts the model training and marker effect estimation, and the trained model cannot predict phenotype well. The other explanation is the marker number, which determines the model’s estimation accuracy [35]. We used 431 299 markers, which is far more than the numbers in previous studies (around 10 000), to make the prediction. Interestingly, the GWAS-associated markers achieved better accuracy than the whole-genome markers (64.1% versus 59.6%), suggesting that it is not the marker number but the number of useful markers that determines GP accuracy for predicted traits. Using machine learning and GWAS to estimate marker effect could increase alfalfa yield prediction accuracy [36].

### The possibility of improving alfalfa using genomic prediction

Because of the outcrossing and self-incompatibility feature of alfalfa, its genotype differs for individuals. Selection was based on the individual in the breeding process, while usage was based on the average performance of lots of plants (population) [37]. If the connection between the selected individuals and the selected population is close, it is practical to use individual plants to predict the whole population’s performance. The GP results showed that using GWAS-associated markers and the SVM linear model could predict the entire population FD (from one individual, predict 15 individuals). Marker selection is one critical step for building the model. We found that 3000 GWAS-associated markers generated the highest accuracy, and more or fewer markers reduced accuracy. Marker selection needs to be considered for building the training model. Besides FD prediction, we extended our model to fall growth, a dormancy-related trait. Generally, accessions with high dormancy have low growth speed in fall after cutting [38]. We used the individual mean and the mean of all phenotypes to represent the complete information on FD. Both kinds of GWAS-associated markers were used to select SNPs. Using the SVM linear model and 3000 markers to make a prediction, the prediction accuracy was similar to the actual situation (59% and 62%). The findings suggested that our model is efficient in predicting FD-related traits. Furthermore, our model-building experience is applicable for trait prediction in alfalfa. The SVM linear model and 3000 GWAS-associated markers are applicable for GP in further research.

The breeding method of alfalfa has been based majorly on recurrent phenotypic selection [37], which relies on population selection of good-performance individuals for further breeding processes. GP selection has a similar selection strategy. For phenotypes that are hard to collect, it is handy to do prediction based on genotypic information. In this study, 176 out of 220 accessions had a fall growth phenotype, but this was not so not for the rest of the accessions. To compare the whole population, we predicted fall growth using FD-related models. Thus, prediction of unknown trait(s) is enabled by using a model for the related traits. Finally, we could predict all critical phenotypes of alfalfa using a GP model.

In conclusion, we combined GP and GWAS and efficiently analyzed the FD of alfalfa using the machine learning method. The results can serve as basic information for GP to help unveil the relationship between genotype and FD phenotype in alfalfa. Application of GP with the machine learning method will benefit the generation of new alfalfa cultivars with different dormancy levels.

## Materials and methods

### Plant materials and phenotyping

The plant materials used in this study consisted of 220 accessions collected from all over the world. These accessions included 55 cultivars, 26 cultivated materials, 95 landraces, 4 breeding materials, 16 wild materials, and 24 uncertain improvement status materials. Among them, 26 accessions were from the database of the Medium Term Library of National Grass Seed Resources of China and the remaining 194 accessions were from the database of the US National Plant Germplasm System (USDA GRIN). The geographic sources of the accessions included China, the USA, Turkey, Afghanistan, Uzbekistan (Tashkent), Spain, Russia, Morocco, France, Argentina, and others (Supplementary Data Table S1).

In October 2017, seeds of the 220 accessions were planted in a greenhouse under conditions of 16 hours day/8 hours night, 22°C and 40% relative humidity. Light intensity (a combination of natural light and incandescent lamps) was ~200 μmol m^−2^ s^−1^. In April 2018 the plantlets were transplanted to the field of the Chinese Academy of Agricultural Sciences (CAAS) in Langfang, Hebei Province, China (39.59°N, 116.59°E). The area has a continental monsoon climate with an average temperature of 11.9°C/year. The coldest month is January, with an average temperature of −4.7°C and the hottest month is July, with an average temperature of 26.2°C. The annual rainfall is 554.9 mm. The soil type is loam soil with a pH value of 7.37 and soil organic matter content is 1.69%. The experimental design was a randomized complete block design with three replications. Every accession containing five plantlets generated from individual seeds was placed 30 cm apart in a single row for one repetition. The accession spacing was 65 cm between rows and columns. To keep uniformity, all plantlets were clipped to a height of 5 cm after transplanting. The alfalfa was cut three times in 2018 (14 June, 28 July, 1 October) and four times in 2019 (10 May, 19 June, 14 August, 19 September). The spring regrowth performance is shown in Supplementary Data Fig. S1. Fall dormancy was the plant regrowth height 1 month after the final clipping (31 October 2018 and 19 October 2019). The tallest stem height (height was measured with a ruler and recorded in centimeters) of one individual accession was considered FD. Plant regrowth height was recorded 2 weeks post-clipping in autumn (1 September 2018 and 29 August 2019). We downloaded fall growth from the GRIN-Global database (https://npgsweb.ars-grin.gov/gringlobal/descriptordetail?id=68081). Fall growth was defined as the plant height on 11–12 October. One hundred and seventy-six accessions had fall growth phenotype data.

The variance of fall dormancy was estimated using a generalized linear model (GLM) as follows:

$$ \begin{align*} \mathrm{Height}&=\mathrm{accession}+\mathrm{ind}\big(\mathrm{accession}\big)+\mathrm{year}+\mathrm{accession}
\ast \mathrm{year}\\&\quad+\mathrm{replicates}+\mathrm{error} \end{align*}$$

All factors were random, and the GLM was performed using PROC GLM (SAS Institute, 2010). The broad-sense heritability (*h*^2^) was computed as follows:

$$ {h}^2={\upsigma}_{\mathrm{g}}^2/\left({\upsigma}_{\mathrm{g}}^2+\frac{\upsigma_{\mathrm{g}\mathrm{y}}^2}{y}+\frac{\upsigma^2}{yir}\right) $$

where ${\upsigma}_{\mathrm{g}}^2$ is the variance of accession, ${\upsigma}_{\mathrm{gy}}^2$ is the interaction variance of accession and year, ${\upsigma}^2$ is the residual variance. The items *y*, *i*, and *r* in the equation refer to the number of years, individuals, and replications, respectively.

### Sequencing and single-nucleotide polymorphism calling

For FD phenotype, five individuals per accession were evaluated with three replicates in a randomized complete block design. An individual with a typical phenotype (phenotype similar to most individuals among the same accession) was selected for sequencing. Young leaves were collected at the early regrowth stage (9 July 2019). Total DNA was extracted using the CWBIO Plant Genomic DNA Kit (CoWin Biosciences, Beijing, China), according to the manufacturer’s protocol. At least 6 μg of genomic DNA from each accession was used to construct a sequencing library following the manufacturer’s instructions (Illumina Inc.). Paired-end sequencing libraries with an insert size of ~300 bp were sequenced on an Illumina NovaSeq 6000 sequencer at Berry Genomics company. The data size of every accession was 10 Gb, and the average Q30 was 85%. The raw data have been uploaded to the National Genomics Data Center (NGDC, https://bigd.big.ac.cn/) under BioProject PRJCA004024 and NCBI Sequence Read Archive with BioProject ID: PRJNA739212. Sequencing data were filtered using Trimmomatic software with default parameters [39]. Paired-end sequencing reads were mapped to the alfalfa reference genome (haploid genome with eight chromosomes [33]) with BWA-MEM using default parameters [40]. SAMtools was used to translate SAM files to BAM files and sort BAM files using default parameters [41].

Picard Tools was used to mark duplicate reads (http://broadinstitute.github.io/picard/), and the Genome Analysis ToolKit was used to correct indels which can be mistaken for SNPs [42]. SAMtools mpileup and VarScan were used to detect SNPs [43]. Furthermore, SNP data were filtered using VCFtools [44] with a missing rate of <10%, a minor allele frequency of >0.05, and a mean read depth >20.

### Association mapping

The Bayesian-information and linkage-disequilibrium iteratively nested keyway (BLINK) method was used to carry out the GWAS [45] for two kinds of FD phenotype (sequencing individuals and mean of 15 individuals). GWAS was conducted using the BLINK C version software. The BLINK method uses iterations to select trait-associated markers. These associated markers were fitted as a covariate for testing the remaining markers [45]. The Manhattan and Q–Q plots of GWAS results were created using the R package qqman [46].

### Genomic prediction with different models

Two machine learning models, SVM regression with the linear kernel (SVM linear) and poly kernel (SVM poly), and three regularization models, Lasso (L1 regularization), ridge regression (L2 regularization) and ElasticNet (combining L1 and L2 regularization), were used as training models. L1 and L2 regularization represent two types of marker effect estimation. The former adapts to a few major QTLs and the latter adapts to many minor-effect QTLs [47]. All five models were used to make FD predictions. Pearson’s correlation coefficient between the predicted and the observed phenotype was assessed as predicted accuracy. The predicted bias was calculated as the regression coefficient between the predicted and the observed phenotype. If a regression coefficient is equal to 1, there is no bias. A regression coefficient greater or smaller than 1 indicates inflated or deflated predictions, respectively [48]. The training and testing populations were split randomly into 80% and 20% genotypes (5-fold cross-validation). This cross-validation procedure was repeated 100 times. Two types of FD phenotype (sequencing individual and mean of 15 individuals) and phenotypes for different years (2018, 2019, and mean of two years) were used to compare model accuracy. As for FD prediction, the GWAS results of sequencing individuals were used to filter markers. The top 100–10 000 associated markers (100, 500, 750, 1000, 2000, 3000, 4000, 5000, and 10 000) were used to build the SVM linear FD predicted model. The mean of 15 individuals’ FD was used as a validated phenotype. FD predicted results were evaluated as optimal associated marker numbers. Fall growth phenotype and GWAS-associated markers of two kinds of phenotype (sequencing individual and mean of 15 individuals) were used to build a new fall growth prediction model. Plant regrowth height phenotype was used as validation phenotype. A customized Python script was written to perform the related analyses. The Python package sklearn was used to build SVM linear, SVM poly, Lasso, ridge regression and ElasticNet models (https://scikit-learn.org/stable/).

## Acknowledgements

We thank the Medium Term Library of National Grass Seed Resources of China and the U.S. National Plant Germplasm System (USDA GRIN) for providing the alfalfa accessions. This work was supported by the National Natural Science Foundation of China (No. 31971758), the breeding forage and grain legumes to increase China’s and EU’s protein self-sufficiency, collaborative research key project between China and EU (2017YFE0111000/EUCLEG 727312), Key Projects in Science and Technology of Inner Mongolia (2021ZD0031), and the China Scholarship Council (201903250068). The funding bodies played no role in the study’s design, the collection, analysis, and interpretation of data, or the writing of the manuscript.

## Author contributions

Q.C.Y. conceived and designed the experiments. J.M.K., R.C.L., M.N.L., and Y.S. planted the alfalfa accessions. F.Z., J.M.K., R.C.L., F.H., X.Q.J., C.F.Y., and X.J.Y. collected the phenotypes. F.Z., J.K., Y.W.W., Z.W., and Z.W.Z. analyzed the data. F.Z., Z.W.Z., Z.W., and Q.C.Y. wrote the paper. All authors read and approved the final manuscript.

## Data availability

All RAD raw sequence data were upload to the National Genomics Data Center (NGDC, https://bigd.big.ac.cn/) under BioProject PRJCA004024 (https://ngdc.cncb.ac.cn/search/?dbId=biosample&q=PRJCA004024&page=1) and NCBI Sequence Read Archive with Bioproject ID: PRJNA739212 (https://www.ncbi.nlm.nih.gov/sra/?term=PRJNA739212). The datasets used and analyzed during the current study are available from the corresponding author on reasonable request.

## Conflict of interest

The authors declare that they have no conflicts of interest.

## Supplementary data

Supplementary data is available at *Horticulture Research* online.

## Supplementary Material

Web_Material_uhac225
