# Supplementary material for: Application of machine learning to explore the genomic prediction accuracy of fall dormancy in autotetraploid alfalfa
Source: Hortic Res. 2022 Oct 7;10(1):uhac225. doi: 10.1093/hr/uhac225 (PMC9832841; doi:10.1093/hr/uhac225)
Supplement: Web_Material_uhac225 [file web_material_uhac225.zip › supplemental_file.docx]

**Supplementary Materials for**

**Application of machine learning to explore the genomic prediction accuracy of fall dormancy in autotetraploid alfalfa**

Fan Zhang^1,2^, Junmei Kang^1^, Ruicai Long^1^, Mingna Li^1^, Yan Sun^3^, Fei He^1^, Xueqian Jiang^1^, Changfu Yang^1^, Xijiang Yang^1^, Jie Kong^1^, Yiwen Wang^4^, Zhen Wang^1^, Zhiwu Zhang^2^*, Qingchuan Yang^1^*

^1^Institute of Animal Science, Chinese Academy of Agricultural Sciences, Beijing, China;

^2^Department of Crop and Soil Sciences, Washington State University, Pullman, WA, USA;

^3^Department of Turf Science and Engineering, College of Grassland Science and Technology, China Agricultural University, Beijing, China.

^4^Melbourne Integrative Genomics, School of Mathematics and Statistics, University of Melbourne, Melbourne, Australia, 3052.

*Correspondence should be addressed to ZZ (Zhiwu.Zhang@wsu.edu) or QY (qchyang66@163.com)


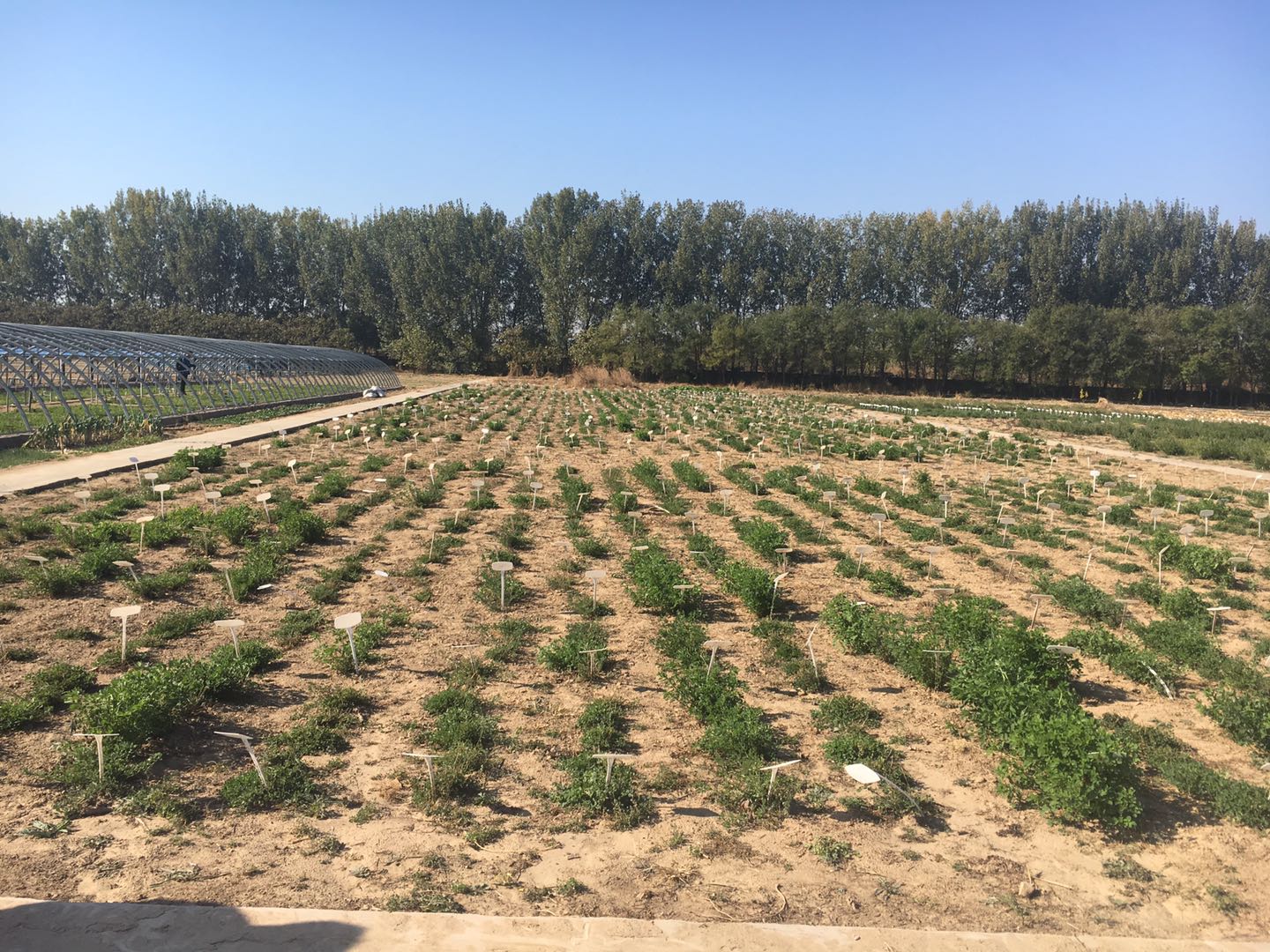


**Figure S1. The spring regrowth performance of 220 accessions.** The regrowth speed was different among accessions.


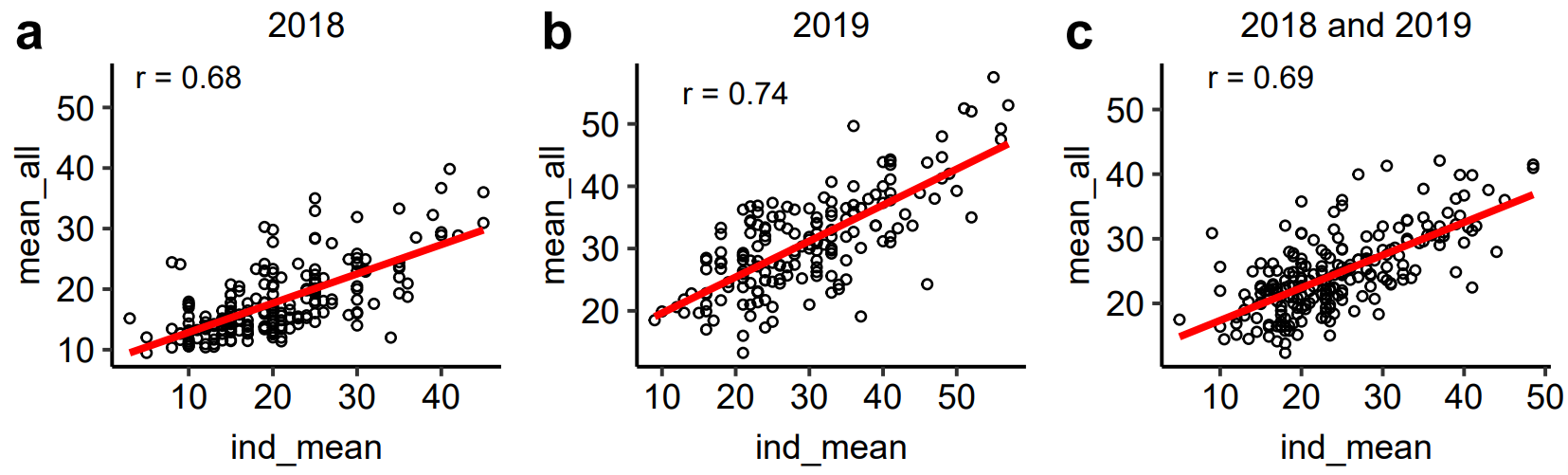


**Figure S2. Correlation of FD between individual mean FD (ind_mean) and mean all FD (mean_all).** Every cycle dot represented one accession. The regression line was drawn with a red line, and Pearson’s correlation coefficient was present at the top left of the figure. a, the correlation information in 2018. b, the correlation information in 2019. c, the correlation information of 2018 and 2019.


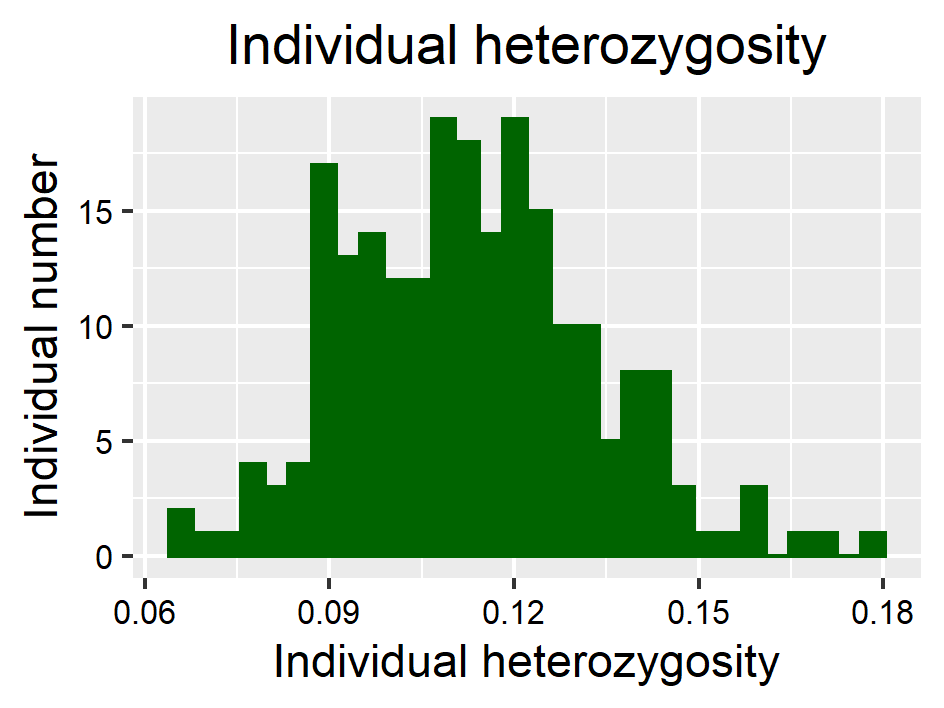


**Figure S3. The individual heterozygosity of 220 accessions.** The mean heterozygosity is 0.11 among 220 accessions. The heterozygosity ranged from 0.06 to 0.18 in 220 accessions.


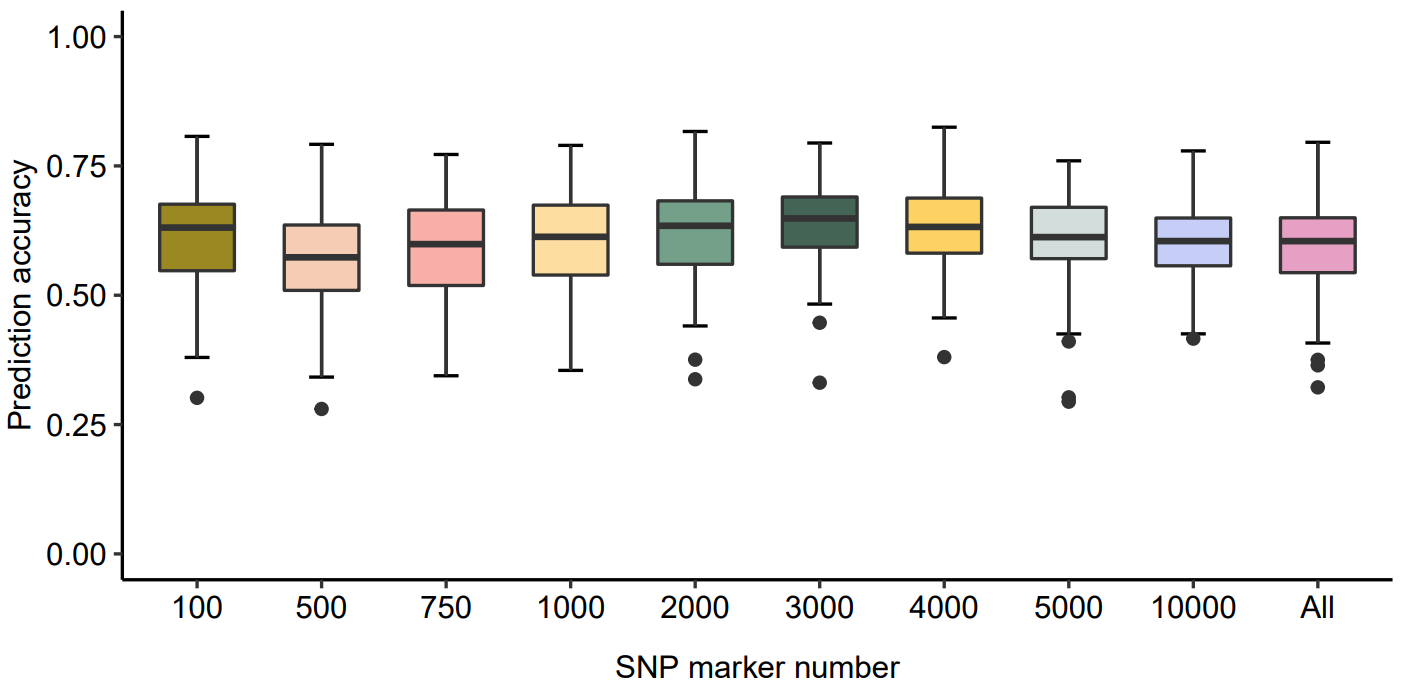


**Figure S4. The box plot of mean all FD prediction accuracy among different numbers of individual mean FD GWAS associated markers.** The top 100 to 10,000 GWAS-associated markers were used to make the prediction. All markers (875,023) were used to check the mean predicted accuracy.
